# All-Aluminum Thin Film Transistor Fabrication at Room Temperature

**DOI:** 10.3390/ma10030222

**Published:** 2017-02-23

**Authors:** Rihui Yao, Zeke Zheng, Yong Zeng, Xianzhe Liu, Honglong Ning, Shiben Hu, Ruiqiang Tao, Jianqiu Chen, Wei Cai, Miao Xu, Lei Wang, Linfeng Lan, Junbiao Peng

**Affiliations:** 1 State Key Laboratory of Luminescent Materialsand Devices, Institute of Polymer Optoelectronic Materials and Devices, South China University of Technology, Guangzhou 510640, China; yaorihui@scut.edu.cn (R.Y.); 201520114219@mail.scut.edu.cn (Z.Z.); 15360452751@163.com (Y.Z.); lxz900618@icloud.com (X.L.); hushiben@foxmail.com (S.H.); 201510102158@mail.scut.edu.cn (R.T.); c.jianqiu@mail.scut.edu.cn (J.C.); c.w01@mail.scut.edu.cn (W.C.); xumiao4049@126.com (M.X.); mslwang@scut.edu.cn (L.W.); lanlinfeng@scut.edu.cn (L.L.); 2 State Key Laboratory of Luminescence and Applications, Changchun Institute of Optics, Fine Mechanics and Physics, Chinese Academy of Sciences, Changchun 130033, China

**Keywords:** thin film transistor, conductor/insulator heterojunction, all-aluminum, room temperature

## Abstract

Bottom-gate all-aluminum thin film transistors with multi conductor/insulator nanometer heterojunction were investigated in this article. Alumina (Al_2_O_3_) insulating layer was deposited on the surface of aluminum doping zinc oxide (AZO) conductive layer, as one AZO/Al_2_O_3_ heterojunction unit. The measurements of transmittance electronic microscopy (TEM) and X-ray reflectivity (XRR) revealed the smooth interfaces between ~2.2-nm-thick Al_2_O_3_ layers and ~2.7-nm-thick AZO layers. The devices were entirely composited by aluminiferous materials, that is, their gate and source/drain electrodes were respectively fabricated by aluminum neodymium alloy (Al:Nd) and pure Al, with Al_2_O_3_/AZO multilayered channel and AlO*_x_*:Nd gate dielectric layer. As a result, the all-aluminum TFT with two Al_2_O_3_/AZO heterojunction units exhibited a mobility of 2.47 cm^2^/V·s and an *I*_on_/*I*_off_ ratio of 10^6^. All processes were carried out at room temperature, which created new possibilities for green displays industry by allowing for the devices fabricated on plastic-like substrates or papers, mainly using no toxic/rare materials.

## 1. Introduction

Metal oxide semiconductors (MOSs) are supposed to be promising materials for thin film transistor (TFT) in displays, which have many favorable properties including high mobility, good uniformity, and electrical stability [1,2]. Furthermore, it is expected that MOS-based devices at room temperature process are compatible with flexible plastic or paper substrate devices [3,4]. Theoretically, MOS-based devices can overcome many obstacles and limitations of the conventional silicon devices, such as a complex process and high cost.

Recently, the attention of researchers has been focused on the novel design devices of nanoscale-stacked materials, which are formed by sequentially depositing different materials in the nanometer scale [5,6,7,8]. However, most of the nanoscale stacked oxide thin film transistors reported a required annealing process to improve the electrical properties. The key issue is the ability to surmount potential barrier from the heterojunction interface scattering carriers. Recently, researches on the semiconductor/insulator multilayers like ZnO/HfO_2_ [9] and ZnO/Al_2_O_3_ [10] used to confine electrons in the potential wells were reported. However, the thermal treatments were still required due to the nature of semiconductors [11] and multilayered structures. Obviously, the thermal treating process is harmful for extending flexible substrates [12], especially for the papers. To solve these problems, we selected aluminum doping zinc oxide (AZO) conductive thin film as one of channel materials, which provided sufficient carriers and helped improving the mobility of the devices without thermal treatment, with the characteristics of high carrier concentration [13], non-toxic [14] and inexpensive [15]. On the contrary, indium gallium zinc oxide (IGZO) [16], indium zinc oxide (IZO) [17], and indium tin zirconium oxide (ITZO) [18], as channel materials for thin film transistors, contain indium element, which is known being toxic and rare in the earth. Moreover, it was reported that the pulsed laser deposition (PLD) produced a flux of energetic ions, which leads to local heating right at the film growth region, playing a similar effect of heat treatment, without imposing a large heat load to the substrate [19].

In this work, we designed three different types of multilayered thin film transistors with AZO conductive layers and Al_2_O_3_ insulating layers to investigate the stacked structure effect of channel layers. We found that the saturation mobility of multilayered TFT rose rapidly when the number of stacked layers increased to four. However, the stacked structure also made the density of defect states increase.

## 2. Experiments

**Figure 1:**
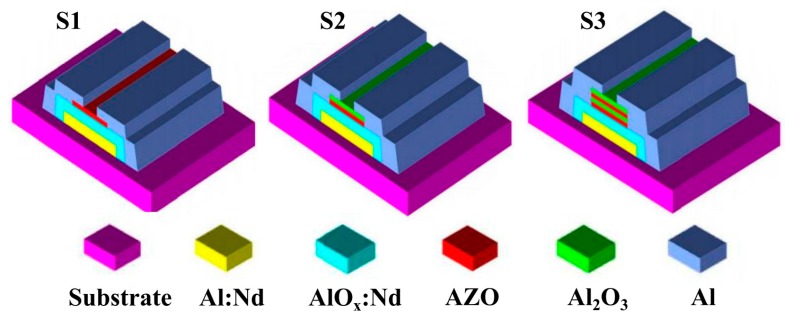
Schematic cross-sectional images of Devices S1, S2, and S3 with different types of channel layer structures.

Figure 1 shows the TFT devices with different channel structures: (i) AZO-TFT, referred as “S1”; (ii) AZO/Al_2_O_3_-TFT, referred as “S2”; and (iii) AZO/Al_2_O_3_/AZO/Al_2_O_3_-TFT, referred as “S3”. A 300-nm-thick Al:Nd alloy (3 wt % of Nd) as gate electrode was deposited on glass substrate by DC magnetron sputtering and patterned by conventional photolithography at room temperature. Subsequently, the gate metal was immersed into the anodizing electrolyte, applied with a voltage of 90 V, forming a 200-nm-thick layer of AlO*_x_*:Nd on the gate surface. AZO and Al_2_O_3_ channel layers were prepared by pulsed laser deposition at room temperature with a basic pressure of 2.0 × 10^−4^ Pa, an O_2_ flow rate of 10 sccm, a pulsing energy of 100 mJ, a repeating rate of 5 Hz, a pulse duration of 10 ns, and a KrF laser wavelength of 248 nm, patterned through the shadow mask. AZO films were all composed of 2 wt % Al_2_O_3_ and 98 wt % ZnO. Al source/drain electrodes with thicknesses of 200 nm were evaporated by Edward evaporation at room temperature. No annealing treatment was adopted during the whole process, and the devices were entirely composed of aluminiferous materials.

The electrical characteristics of TFTs were measured by a semiconductor parameter analyzer (Agilent 4155 C, Santa Clara, CA, USA) under ambient condition.

## 3. Results and Discussion

**Figure 2:**
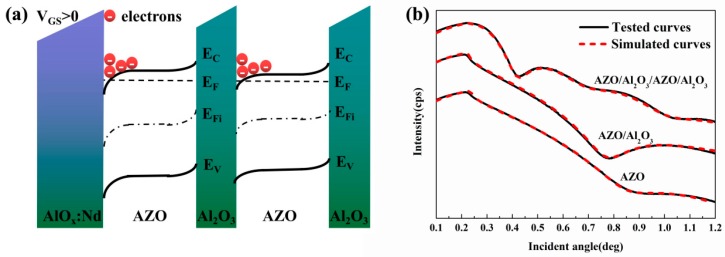
(**a**) Energy band structure and electrical effect of AZO/Al_2_O_3_/AZO/Al_2_O_3_ stacked channel layers; (**b**) X-ray reflectivity (XRR) measurements obtained from AZO single layer, AZO/Al_2_O_3_ bilayer, and AZO/Al_2_O_3_/AZO/Al_2_O_3_ multilayers.

As shown in Figure 2a, in the AZO/Al_2_O_3_ heterojunction structure, because of the high conduction band offset between AZO and Al_2_O_3_, electrons can be accumulated in a potential well of AZO [7]. Thus, along the in-plane direction, the high electron movement was expected to be induced by the AZO/Al_2_O_3_ multilayers, due to the two dimension electron transfer formed in the interfaces between AZO and Al_2_O_3_. Moreover, the channel current in the multilayered structure was formed through both in-plane and out-of-plane directions. The out-of-plane current strongly depends on the thickness of the barrier layers since the carriers can migrate along the vertical direction in the multi-structures through direct tunneling, which requires that the Al_2_O_3_ barrier layers should be ultrathin. The tested curves and simulated curves of the X-ray reflectivity (XRR, EMPYREAN, PANalytical, Almelo, The Netherlands) measurement are shown in Figure 2b. The result shows that the thickness of AZO films is between 2.6 and 3.8 nm, with a roughness of 0.57–0.92 nm; and the thickness of Al_2_O_3_ films ranges from 2.1 to 2.6 nm, with a roughness from 0.41 to 0.83 nm. The experiment indicated has acquired smooth and ultrathin nano-multilayers. 

**Figure 3:**
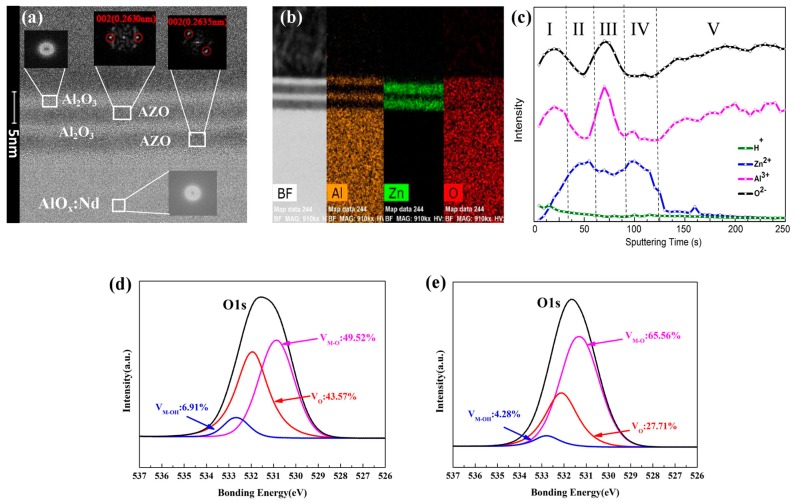
(**a**) Cross-sectional high-resolution transmission electron microscopy *(*HRTEM) image of AZO/Al_2_O_3_/AZO/Al_2_O_3_ channel layers in Device S3 and (**b**) the results of Al, Zn, O distribution detected by energy-dispersive X-ray spectroscope (EDS) mapping scan; (**c**) Time of flight secondary ion mass spectrometry (TOF-SIMS) results of H^+^, Zn^2+^, Al^3+^, and O^2−^ ions for Device S3: Region I and III corresponds with Al_2_O_3_ layers, Region II and IV corresponds with AZO layers, and Region V corresponds with the anodized AlO*_x_*:Nd gate insulator. X-ray photoelectron spectra (XPS) for the O1s region of (**d**) the PLD grown Al_2_O_3_ layer and (**e**) the anodized AlO*_x_*:Nd gate insulator.

Figure 3a shows the cross-sectional high-resolution transmission electron microscopy *(*HRTEM, JEM-2100F, JEOL, Akishima, Tokyo, Japan) image of AZO/Al_2_O_3_/AZO/Al_2_O_3_ channel layers in Device S3, and smooth interfaces between ~2.7-nm-thick AZO and ~2.2-nm-thick Al_2_O_3_ layers can be observed, consistent with the XRR results. It indicates that the ultrathin multilayers were well-deposited by the PLD method. In addition, the electron diffraction patterns of AZO/Al_2_O_3_ multilayers manifested the structure of crystalline/amorphous. Both AZO layers grown on the anodized AlO*_x_*:Nd gate insulator and PLD prepared Al_2_O_3_ layer showed the similar diffraction plane of (002) (common in as-deposited PLD grown AZO or ZnO as reported [20,21]), suggesting that the AZO/Al_2_O_3_ heterojunction unit can be well repeated by PLD method without the effect of different underlayers. Moreover, there were no obvious structural differences between the anodized AlO*_x_*:Nd gate insulator and PLD grown Al_2_O_3_ layers. 

The results of Al, Zn, O distribution detected by energy-dispersive X-ray spectroscope (EDS, Bruker, Adlershof, Berlin, Germany) mapping scan are shown in Figure 3b. Through EDS mapping scan, an obvious diffusion of Zn element from AZO layers into Al_2_O_3_ layers was found, while which was rare in the anodized AlO*_x_*:Nd gate insulator. It was verified by the results of time of flight secondary ion mass spectrometry (TOF-SIMS, PHI TRIFT-II, Physical Electronics, Minneapolis and Saint Paul, MN, USA), which is shown in Figure 3c. This phenomenon was possibly caused by the strong adsorption of Zn atoms in the Al_2_O_3_ layers, because of the high content of oxygen vacancies, enlarging the pore mouth of the ultrathin Al_2_O_3_ film and increasing the adsorption ability. As shown by the X-ray photoelectron spectra (XPS, ESCALAB 250Xi, Thermo Fisher Scientific, Waltham, MA, USA) for O1s region in Figure 3d,e, the content of oxygen vacancies of PLD grown Al_2_O_3_ layer is much higher than the anodized AlO*_x_*:Nd gate insulator, which can explain their different degrees of Zn diffusion. Meanwhile, in Figure 3c, we can also observe a strong and sharp peak of Al^3+^ in Region III, which implies an inward gathering of Al^3+^ ions in the Al_2_O_3_ layers. It may be due to the positive charge repulsion by Zn^2+^ ions as they diffused from both sides of Region II and Region IV.

**Figure 4:**
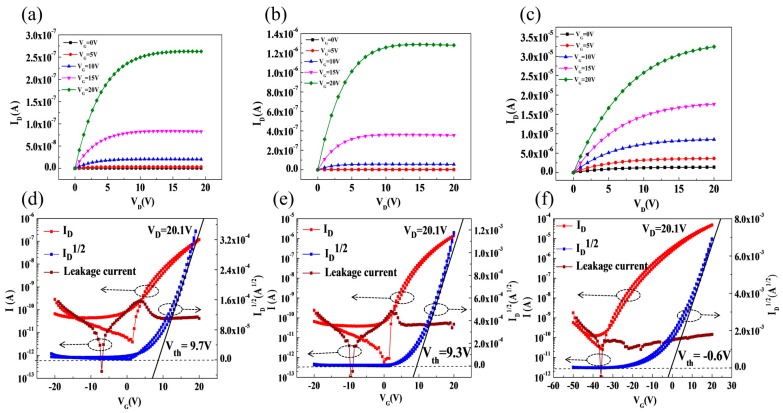
Output characteristics (**a**–**c**) and transfer characteristics (**d**–**f**) of devices: (**a**,**d**) for S1; (**b**,**e**) for S2 and (**c,f**) for S3.

Figure 4a–f shows the output and transfer characteristics of the three devices with different structures of channel layers and the relevant data is listed in Table 1. The channel width/length (W/L) of all the devices was 1000/300 μm and the capacitance used to calculate mobility was 38 nF/cm^2^. Compared with Device S1, Device S2 with an ultrathin Al_2_O_3_ barrier layer exhibited higher saturation mobility (μ_sat_) and on-state current (*I*_on_), which indicates that the AZO/Al_2_O_3_ stacked structure can improve the electrical performance of devices. It showed a similar tendency compared with other passivated TFTs from the literature [22,23,24]. However, in those researches, the passivation layers were thick (100–300 nm) and required heat treatment for preparation, which were unfavorable for ultrathin and flexible displays. Moreover, it is worth noticing that the saturation mobility of Device S3 is one order higher than Device S2, which was significantly promoted by the increase of channel paths [25].

The sub-threshold swing (SS) value is related to the total defect density from the bulk channel layer and the interface between the channel and dielectric layers [26]. The SS value is defined at the minimum value of (dlog(*I*_DS_)/dV_GS_))^−1^. With the increasing number of stacked layers, the SS value elevated, according to Table 1. It indicates that stacked structure caused the increase of defects in the interfaces or bulk of channel layers [27], which was possibly attributed to the diffusion of Zn in the ultrathin Al_2_O_3_ barrier layers, as shown by the results of HRTEM and TOF-SIMS in Figure 3b,c. As the number of interfaces increased, the effect of Zn diffusion became more significant, which should be well concerned. Additionally, the serious negative *V*_on_ in S3 was possibly due to the great number of conduction electrons trapped in the interfaces between the layers, which were increased by the ion bombardment on the surfaces of underlayers during the PLD process as well. This phenomenon should be worked out in our further research.

Generally, the positive *V*_th_ shift of oxide TFTs is considered related to the charge trapping mechanism and extra negative charge capture by the adsorption of oxygen molecules in the back channel [28]. As shown in Figure 4, both devices S1 and S2 suffered a large positive shift in *V*_th_ with Δ*V*_th_ of 9.7 and 9.3 V, respectively. However, the Δ*V*_th_ of Device S3 reduced significantly to −0.6 V. It could be due to the higher carrier concentration with the increasing number of channel paths, and the increase of defect sites like oxygen vacancies in the interface between the upper AZO layer and under Al_2_O_3_ layer, as well. In addition, the reduction of Δ*V*_th_ in the devices with an increasing number of stacked layers also indicates that the barrier layers are able to help restrain the back channel effect to some degree [29].

## 4. Conclusions

In summary, three different types of all-aluminum thin film transistors were fabricated at room temperature. The smooth interfaces between ~2.7-nm-thick AZO layers and ~2.2-nm-thick Al_2_O_3_ layers were observed through the HRTEM images, consistent with the XRR results. The device with AZO/Al_2_O_3_/AZO/Al_2_O_3_ multilayered channels showed a saturation mobility of 2.47 cm^2^/V·s and an on-to-off current ratio of 1.92 × 10^6^. Ultrathin alumina (Al_2_O_3_) insulating layer deposited on the surface of aluminum doping zinc oxide (AZO) conductive layers can effectively confine the electron in potential well of AZO. The parallel channel paths can significantly increase the channel current and improve mobility.

It is worth mentioning that all processes were carried out at room temperature, which allows for the devices fabricated on plastic-like substrates or papers. Therefore, it is expected that the all-aluminum TFT with multilayered structure will create a new opportunity for an eco-friendly industry of flexible and wearable displays.

## Figures and Tables

**Table 1:** Device parameters extracted from the transfer curves in Figure 2, including on-to-off current ratio (*I*_on_/*I*_off_), sub-threshold swing (SS), saturation mobility (μ_sat_), and threshold voltage (*V*_th_).

Device	*I*_on_/*I*_off_	SS (V/Decade)	μ_sat_ (cm^2^/V·s)	*V*_th_ (V)
S1	3.02 × 10^4^	0.86	0.04	9.7
S2	7.47 × 10^4^	1.53	0.50	9.3
S3	1.92 × 10^6^	2.34	2.47	−0.6

## References

[1] Kenji N., Ohta H., Takagi A., Kamiya T., Hirano M., Hosono H. (2004). Room-temperature fabrication of transparent flexible thin-film transistors using amophous oxide semiconductors. Nature.

[2] Jung S.W., Chae S.S., Park J.H., Oh J.Y., Bhang S.H., Baik H.K., Lee T.I. (2016). Microscale soft patterning for solution processable metal oxide thin film transistors. ACS Appl. Mater. Interfaces.

[3] Yabuta H., Zhu B., Ast D.G., Greene R.G., Thompson M.O. (2006). High-mobility thin-film transistor with amorphous InGaZnO_4_ channel fabricated by room temperature RF-magnetron sputtering. Appl. Phys. Lett..

[4] Park J.S., Maeng W., Kim H., Park J. (2012). Review of recent developments in amorphous oxide semiconductor thin-film transistor devices. Thin Solid Films.

[5] Homola T., Buršíková V., Ivanova T.V., Souček P., Maydannik P.S., Cameron D.C., Lackner J.M. (2015). Mechanical properties of atomic layer deposited Al_2_O_3_/ZnO nanolaminates. Surf. Coat. Technol..

[6] Nayak P.K., Wang Z., Anjum D.H., Hedhili M.N., Alshareef H.N. (2015). Highly stable thin film transistors using multilayer channel structure. Appl. Phys. Lett..

[7] Ahn C.H., Senthil K., Cho H.K., Lee S.Y. (2013). Artificial semiconductor/insulator superlattice channel structure for high-performance oxide thin-film transistors. Sci. Rep..

[8] Lee S., Hwang C., Pi J., Yang J., Oh H., Cho S.H., Cho K., Chu H.Y. (2014). Characterization of amorphous multilayered ZnO-SnO_2_ heterostructure thin films and their field effect electronic properties. Appl. Phys. Lett..

[9] Ahn C.H., Cho H.K., Kim H. (2015). Carrier confinement effect-driven channel design and achievement of robust electrical/photostability and high mobility in oxide thin-film transistors. J. Mater. Chem. C.

[10] Park J.H., Alshammari F.H., Wang Z., Alshareef H.N. (2016). Interface engineering for precise threshold voltage control in multilayer-channel thin film transistors. Adv. Mater. Interfaces.

[11] Ahn C.H., Bo H.K., Kim H., Cho H.K. (2011). Improved electrical stability in the Al doped ZnO thin-film-transistors grown by atomic layer deposition. J. Electrochem. Soc..

[12] Hua X., Luo D., Li M., Xu M., Zou J., Tao H., Lan L., Wang L., Peng J., Cao Y. (2014). A flexible AMOLED display on the PEN substrate driven by oxide thin-film transistors using anodized aluminium oxide as dielectric. J. Mater. Chem. C.

[13] Prabhakar T., Dai L., Zhang L., Yang R., Li L., Guo T., Yan Y. (2014). Effects of growth process on the optical and electrical properties in Al-doped ZnO thin films. J. Appl. Phys..

[14] Kusayanagi M., Uchida A., Oka N., Jia J., Nakamura S., Shigesato Y. (2014). Al-doped ZnO films deposited on a slightly reduced buffer layer by reactive dc unbalanced magnetron sputtering. Thin Solid Films.

[15] Hagendorfer H., Lienau K., Nishiwaki S., Fella C.M., Kranz L., Uhl A.R., Jaeger D., Luo L., Gretener C., Buecheler S. (2014). Highly transparent and conductive ZnO: Al thin films from a low temperature aqueous solution approach. Adv. Mater..

[16] Zan H., Yeh C.C., Meng H.F., Tsai C.C., Chen L.H. (2012). Achieving high field-effect mobility in amorphous indium-gallium-zinc oxide by capping a strong reduction layer. Adv. Mater..

[17] Seo J., Bae B. (2014). Improved electrical performance and bias stability of solution-processed active bilayer structure of indium zinc oxide based TFT. ACS Appl. Mater. Interfaces.

[18] Jia J., Torigoshi Y., Kawashima E., Utsuno F., Yano K., Shigesato Y. (2015). Amorphous indium-tin-zinc oxide films deposited by magnetron sputtering with various reactive gases: Spatial distribution of thin film transistor performance. Appl. Phys. Lett..

[19] Rembert T., Battaglia C., Anders A., Javey A. (2015). Room temperature oxide deposition approach to fully transparent, all-oxide thin-film transistors. Adv. Mater..

[20] Gupta M., Chowdhury F.R., Barlage D., Mosnier J.-P. (2013). Optimization of pulsed laser deposited ZnO thin-film growth parameters for thin-film transistors (TFT) application. Appl. Phys. A.

[21] Inguva S., Vijayaraghavan R.K., McGlynn E., Mosnier J.-P. (2015). Highly transparent and reproducible nanocrystalline ZnO and AZO thin films grown by room temperature pulsed-laser deposition on flexible Zeonor plastic substrates. Mater. Res. Express.

[22] An S., Mativenga M., Kim Y., Jang J. (2014). Improvement of bias-stability in amorphous-indium-gallium-zinc-oxide thin-film transistors by using solution-processed Y_2_O_3_ passivation. Appl. Phys. Lett..

[23] Choi S.H., Han M.K. (2012). Effect of deposition temperature of SiOx passivation layer on the electrical performance of a-IGZO TFTs. IEEE Electron Device Lett..

[24] Wu J., Chen Y., Zhou D., Hu Z., Xie H., Dong C. (2015). Sputtered oxides used for passivation layers of amorphous InGaZnO thin film transistors. Mater. Sci. Semicond. Process..

[25] Lin Y., Faber H., Labram J.G., Stratakis E., Sygellou L., Kymakis E., Hastas N.A., Li R., Zhao K., Amassian A. (2015). High electron mobility thin-film transistors based on solution-processed semiconducting metal oxide heterojunctions and quasi-superlattices. Adv. Sci..

[26] Fortunato E., Barquinha P., Martins R. (2012). Oxide semiconductor thin-film transistors: A review of recent advances. Adv. Mater..

[27] Calatayud M., Markovits A., Menetrey M., Mguig B., Minot C. (2003). Adsorption on perfect and reduced surfaces of metal oxides. Catal. Today.

[28] Nomura K., Kamiya T., Hirano M., Hosono H. (2009). Origins of threshold voltage shifts in room-temperature deposited and annealed a-In-Ga-Zn-O thin-film transistors. Appl. Phys. Lett..

[29] Byungki R., Noh H., Choi E., Chang K.J. (2010). O-vacancy as the origin of negative bias illumination stress instability in amorphous In-Ga-Zn-O thin film transistors. Appl. Phys. Lett..

